# Evaluation and validation of laboratory procedures for the surveillance of ESBL-, AmpC-, and carbapenemase-producing *Escherichia coli* from fresh meat and caecal samples

**DOI:** 10.3389/fmicb.2023.1229542

**Published:** 2023-08-09

**Authors:** Rene S. Hendriksen, Lina M. Cavaco, Beatriz Guerra, Valeria Bortolaia, Yvonne Agersø, Christina Aaby Svendsen, Hanne Nørgaard Nielsen, Jette Sejer Kjeldgaard, Susanne Karlsmose Pedersen, Mette Fertner, Henrik Hasman

**Affiliations:** ^1^National Food Institute, Technical University of Denmark (DTU Food), European Union Reference Laboratory for Antimicrobial Resistance, Research Group for Global Capacity Building, Kgs. Lyngby, Denmark; ^2^Department for Bacteria, Parasites and Fungi, Statens Serum Institut, Reference Laboratory for Antimicrobial Resistance, Copenhagen, Denmark; ^3^European Food Safety Authority, Parma, Italy; ^4^Department of Veterinary and Animal Sciences, University of Copenhagen University, Copenhagen, Denmark

**Keywords:** extended-spectrum beta-lactamase, carbapenemase, isolation method, *Escherichia coli*, protocol, surveillance, European Union

## Abstract

**Introduction:**

Extended-spectrum β-lactamase- (ESBL) and AmpC- β-lactamase-producing *Enterobacterales* are widely distributed and emerging in both human and animal reservoirs worldwide. A growing concern has emerged in Europe following the appearance of carbapenemase-producing *Escherichia coli* (*E. coli*) in the primary production of food animals. In 2013, the European Commission (EC) issued the Implementing Decision on the monitoring and reporting of antimicrobial resistance in zoonotic and commensal bacteria. The European Union Reference Laboratory for Antimicrobial Resistance (EURL-AR) was tasked with providing two laboratory protocols for samples derived from meat and caecal content, respectively, for the isolation of ESBL- and AmpC-producing *E. coli* (part 1) and carbapenemase-producing (CP) *E. coli* (part 2). In this study, we describe the current protocols, including the preparatory work for the development.

**Methods:**

Up to nine laboratory procedures were tested using minced meat as the matrix from beef, pork, and chicken as well as six procedures for the caecal content of cattle, pigs, and chicken. Variables included sample volume, pre-enrichment volume, pre-enrichment broth with and without antimicrobial supplementation, and incubation time/temperature. The procedures were evaluated against up to nine *E. coli* strains harboring different AMR genes and belonging to the three β-lactamase groups.

**Results and discussion:**

The laboratory procedures tested revealed that the most sensitive and specific methodologies were based on a Buffered Peptone Water pre-enrichment of 225 ml to 25 g or 9 ml to 1 g for minced meat and caecal content, respectively, incubated at 37°C overnight, followed by inoculation onto MacConkey agar supplemented with 1 mg/L cefotaxime for detecting ESBL- and AmpC-producing *E. coli* and Chrom ID SMART (Chrom ID CARBA and OXA) for CP *E. coli*, incubated overnight at 37 and 44°C, respectively. We provided two isolation protocols for the EU-specific monitoring of ESBL- and AmpC- producing *E. coli* (part 1) and CP *E. coli* (part 2) from fresh meat (protocol 1) and caecal (protocol 2) samples, which have been successfully implemented by all EU Member States for the monitoring period 2014–2027 (EU 2020/1729).

## Introduction

Extended-spectrum β-lactamase- (ESBL) and AmpC-β-lactamase-producing *Enterobacterales* are widely distributed and emerging in both human and animal reservoirs worldwide (Madec et al., 2017; Mughini-Gras et al., 2019; Dantas and Ferreira, 2020; Aworh et al., 2022). During the last decade, a growing concern has emerged in Europe following the appearance of carbapenemase-producing (CP) *Escherichia coli* (*E. coli*) and *Salmonella infantis*, harboring the *bla*_VIM − 1_ gene isolated from German pigs and chickens in 2011 (Fischer et al., 2012, 2013, 2017; Guerra et al., 2014; Borowiak et al., 2017; Irrgang et al., 2017, 2019; Madec et al., 2017). Since then, several other examples of CP *Enterobacterales* (CPE) have emerged in primary production in Europe, exemplified by *E. coli* isolated in 2015 from meat products at retail in Belgium harboring *bla*_VIM − 1_ (Garcia-Graells et al., 2020), *E. coli* isolated from poultry and poultry meat in Romania in 2016 harboring *bla*_OXA − 48_-like (*bla*_OXA − 162_) (Bortolaia et al., 2021), *E. coli* in Italy in 2019 harboring *bla*_NDM − 4_ (Diaconu et al., 2020), *E. coli* in pigs in Germany in 2019 harboring *bla*_OXA − 48_ and *bla*_GES − 5_ (Irrgang et al., 2020b), and *E. coli* in broilers in Austria in 2020 harboring *bla*_VIM − 1_ (European Food Safety Authority European Centre for Disease Prevention Control, 2023). More recent examples include *E. coli* isolated from fattening pigs harboring *bla*_OXA − 48_ and from a veal calf and fattening pigs in Italy in 2021 harboring *bla*_OXA − 181_ (Carfora et al., 2022; European Food Safety Authority European Centre for Disease Prevention Control, 2023), as well as *E. coli* from fattening pigs in Czechia and *E. coli* from fattening pigs and calves in Hungary harboring *bla*_NDM − 5_ (European Food Safety Authority European Centre for Disease Prevention Control, 2023). Similar findings have also been reported to be emerging outside Europe, in China (Li et al., 2019; Shi et al., 2021), Egypt (Hamza et al., 2016), Australia, and India (Kock et al., 2018).

One of the many drivers behind the emergence of ESBL, AmpC- β-lactamase, and CPE is the likely transmission by horizontal gene transfer, which persists among humans and in the primary production of animals, such as broilers, in the EU (Carattoli, 2008; Mughini-Gras et al., 2019).

The World Health Organization (WHO), the World Organization for Animal Health (WOAH), and the European Medicine Agency (EMA) have, thus, all issued lists of critically important antimicrobial agents for human and veterinary medicine (https://www.ema.europa.eu/en/documents/report/categorisation-antibiotics-european-union-answer-request-european-commission-updating-scientific_en.pdf). Both lists define 3rd and 4th generation cephalosporins, as well as carbapenems, as critically important antimicrobials. Hence, the surveillance of antimicrobial resistance against these antimicrobials in sources outside the human reservoir, e.g., in the food chain, is crucial in early warning systems to enable control and prevent further spread to the public.

Striving toward a harmonized and standardized monitoring of antimicrobial resistance among food and food-producing animals in the EU, the European Commission (EC) on 12 November 2013 issued the Implementing Decision on the monitoring and reporting of antimicrobial resistance in zoonotic and commensal bacteria (European Union, 2013), which was repealed in 2021 by the Implementing Decision (EU) 2020/1729 of 17 November 2020 (European Union, 2020). These regulations laid down rules on the specific monitoring of ESBL-, AmpC-, and CP *E. coli* from meat and caecal samples originating from cattle, pigs, and poultry, stipulating that all member states were obliged to follow the protocol of the European Union Reference Laboratory for Antimicrobial Resistance (EURL-AR). Thus, the EURL-AR was tasked with providing a laboratory protocol for the isolation of ESBL-, AmpC-, and CP *E. coli* from meat and caecal samples (https://www.eurl-ar.eu/protocols.aspx).

In this study, we describe the two European Union Reference Laboratory for Antimicrobial Resistance (EURL-AR) protocols, including parts 1 and 2, used in the monitoring and reporting of antimicrobial resistance in zoonotic and commensal bacteria according to the Implementing Decisions 2013/652/EU and 2020/1729, including the preparatory work for the development.

## Methods

### Sample description

All samples of minced beef, pork, and chicken meat originated from Denmark and were purchased as fresh meat directly from retail supermarkets and transported to the National Food Institute, Technical University of Denmark (DTU Food) in Denmark in polystyrene boxes to maintain the cold chain. Subsequently, the minced meat was stored at 4°C in refrigerators before further processing.

Caecal samples (chicken) and caecal content (pig and cattle) were obtained from Danish slaughterhouses taking part in the EU monitoring. The caecal samples were collected directly from the slaughterhouses and transported to DTU Food in polystyrene boxes to maintain the cold chain. The caecal samples and content were stored at 4°C in refrigerators before further processing, including extracting the caecal content from chickens and pooling five per batch.

### Test isolates

When developing the protocols of meat (protocol 1) and caecal content (protocol 2) samples to detect ESBL- and AmpC-producing *E. coli* (part 1) and CP *E. coli* (part 2), the following resistance genes were included in the test panel. *E. coli* isolates harboring relevant and common genes conferring resistance to 3rd generation cephalosporins and carbapenems were used in validation experiments as positive controls: *bla*_CTX − M−1_ (0412004714_F1), *bla*_SHV − 12_ (0412055161_F131), *bla*_TEM − 52_ (7633094_7), and *bla*_CMY − 2_ (0412056488_F191), all part of DANMAP 2012 (DANMAP, 2012). To detect CP *E. coli*, representative strains carrying *bla*_VIM − 1_ (R178) (Fischer et al., 2012), *bla*_NDM − 1_ (#271) (Poirel et al., 2011), *bla*_OXA − 48_ (KMU AUH), and *bla*_KPC − 2_ (17/11 RKI) were also included in the test panel (Table 1). The *E. coli* ATCC 25922 was used as the susceptible quality control (QC) strain.

**Table 1:** MIC (mg/L) determination and antimicrobial resistance genes of the test strains used for the spiking experiments.

**Target gene**	**Strain no**	**Ampicillin**	**Temocillin**	**Cefoxitin**	**Cefotaxime**	**Cefotaximeclavulanic acid**	**Ceftazidime**	**Ceftazidime/clavlanic acid**	**Cefepime**	**Ertapenem**	**Imipenem**	**Meropenem**
*bla*_CTX − M−1_	0412004714_F1	>32	4	16	32	0.12/4	2	0.25/4	8	0.12	≤ 0.12	≤ 0.03
*bla*_SHV − 12_	0412055161_F131	>32	8	8	4	≤ 0.06/4	32	≤ 0.12/4	1	≤ 0.015	0.25	≤ 0.03
*bla*_TEM − 52_	7633094_7	>32	8	16	8	0.12/4	8	≤ 0.12/4	1	16	≤ 0.12	≤ 0.03
*bla*_CMY − 2_	0412056488_F191	>32	4	64	8	8/4	8	8	0.25	0.06	0.12	≤ 0.03
*bla*_VIM − 1_	R178	>32	32	32	16	16/4	64	32/4	4	0.06	0.5	0.12
*bla*_NDM − 1_	271	>32	>128	>64	>64	>64/4	>128	>128/4	>32	>2	8	>16
*bla*_OXA − 48_	KMU AUH	>32	128	>64	32	32/4	32	32/4	16	>2	1	4
*bla*_KPC − 2_	17/11 RKI	>32	16	>64	64	32/4	16	8/4	8	>2	1	2
Quality Control	ATCC 25922	4	16	2	≤ 0.25	0.12/4	≤ 0.25	0.25/4	≤ 0.12/4	≤ 0.015	≤ 0.12	≤ 0.03

### Validation experiments for detecting specific ESBL- and AmpC-producing *E. coli* (part 1) — Rationale for the initial set of validation experiments

The final protocols should detect bacteria with reduced susceptibility to 3rd generation cephalosporins (part 1) present in meat and caecal samples. Therefore, a broad selection of *E. coli* isolates carrying various genes related to this reduced susceptibility (including carbapenemases) was included in the first part of the validation experiments.

### Spiking procedures of the minced meat samples

From nine batches of 1,500 g retailed minced meat, one batch per nine *E. coli* isolates was divided into six portions of 190 g and six portions of 10 g. The different portions were labeled with the isolate number and with one of the following concentrations: 0, 0.1, 1, 10, 100, and 1,000 CFU/g. One 10 g sample labeled 0 CFU/g for each of the nine 1,500 g retailed minced meat samples was selected as the negative control to ensure that the meat was not already contaminated with an ESBL-producing, AmpC-producing, and CP organism (CPO). The remaining five 10 g samples of each of the nine 1,500 g retailed minced meat samples were spiked individually with one of the nine *E. coli* test strains. The samples were spiked with 0.9% NaCl suspensions containing each of the nine individual *E. coli* test strains to contain 0.1, 1, 10, 100, and 1,000 CFU/g per final weight of 200 g. The lowest dilution step of 0.1 CFU/g was included to ensure an extra dilution in case the experiment did not provide a precise CFU count. In brief, the spiking was prepared as follows: individual *E. coli* test strains were cultured overnight (o/n) at 37°C in 10 ml Luria-Bertani (LB) broth (Sigma-Aldrich) supplemented with 1 mg/L cefotaxime, except for the susceptible control strain (*E. coli* ATCC 25922) and the *bla*_OXA − 48_ strain. The o/n cultures were cooled on ice for 30 min before centrifugation (8,000 g, 10 min) in 10 ml cold 0.9% NaCl. Subsequently, the optical density (OD) was adjusted to OD600 = 0.25 by using 1–4 ml of saline as a starting point (dilution 0). Further serial 10-fold dilutions (10^−2^ to 10^−6^) were prepared from the stock by transferring 1 ml into 9 ml cold 0.9% NaCl and thoroughly mixing it for 20 s between each dilution. After preparing the dilutions, the CFU was assessed in dilutions of 10^−4^, 10^−5^, and 10^−6^ in triplicates. Briefly, 100 μl of each dilution was plated onto LB agar plates (nine plates in total) and incubated at 37°C o/n. Subsequently, the CFUs were enumerated and recorded.

The previously prepared suspensions were used for spiking by mixing 2 mL of different dilutions of pre-adjusted bacterial suspensions (final dilutions between 10^−3^ and 10^−7^ containing between 20 and 2 × 10^5^ bacteria per 2 mL) in 200 g of meat samples to obtain meat samples with the intended CFU count per gram.

The 10 g spiked minced meat samples were homogenized using a stomacher with the corresponding 190 g minced meat samples to make up the final weight of 200 g.

### Minced beef

**Figure 1:**
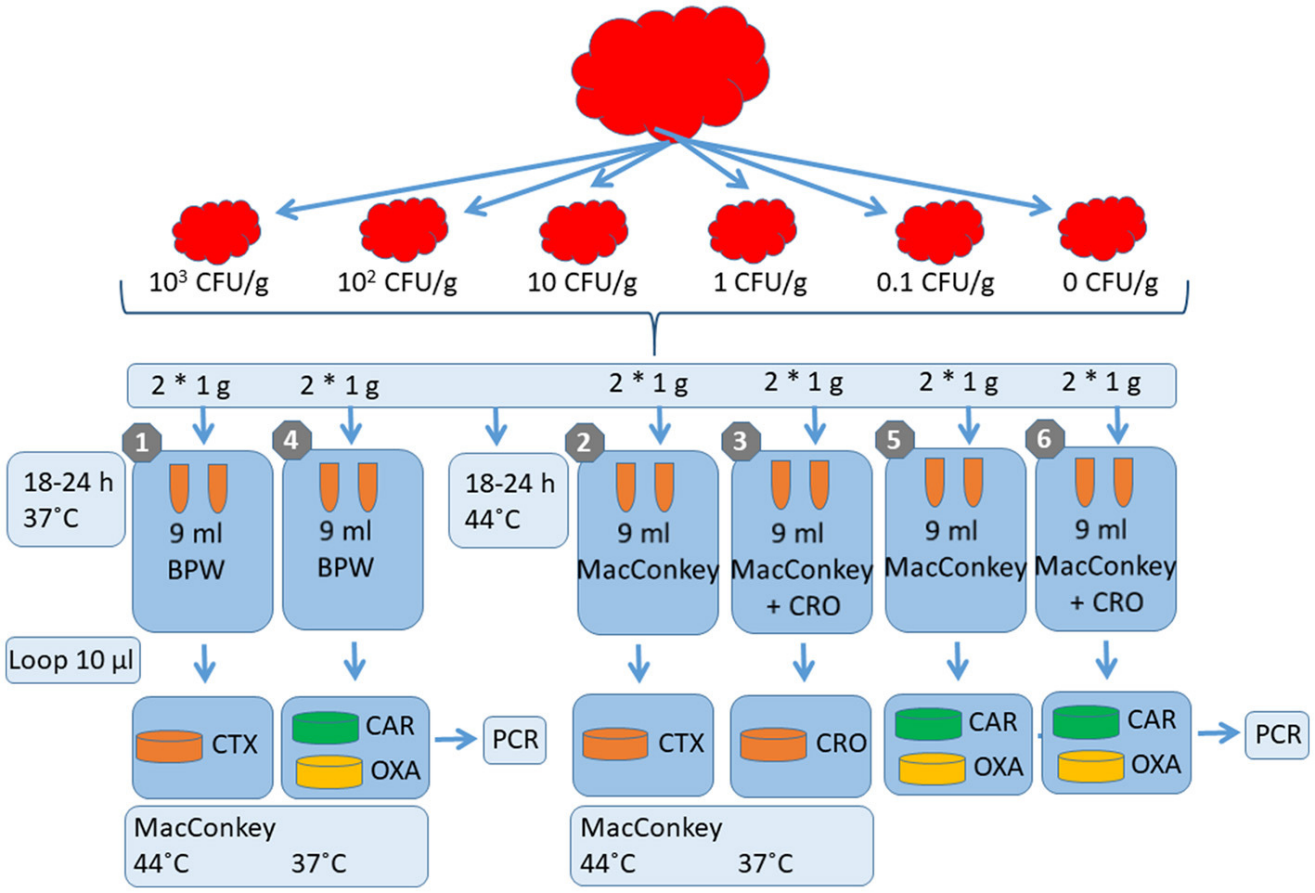
Validation scheme for the comparison of the different detection methods on minced meat content. CFU/g, colony-forming unit per Gram; g, Gram; C, Celsius; h, hour; ml, milliliter; BPW, Buffered Peptone Water; CAR, ChromID CARBA medium; OXA, ChromID OXA media; MacConkey, MacConkey broth or agar; CTX, cefotaxime; CRO, ceftriaxone; PCR, Polymerase chain reaction.

Initially, a full study design was prepared using minced beef as the matrix, which included six laboratory procedures (MM-1 to MM-6) evaluated against all nine *E. coli* strains producing various types of 3rd-generation cephalosporinases or carbapenemases. The initial design was based on the recommendation from the European Food Safety Authority's (EFSA) Scientific Opinion “on the public health risks of bacterial strains producing extended-spectrum β-lactamases and/or AmpC β-lactamases in food and food-producing animals” (EFSA Panel on Biological Hazards, 2011). In this study, the initial six laboratory procedures (MM-1 to MM-6) were conducted in duplicates and varied in terms of (1) pre-enrichment broths and associated incubation temperatures, (2) selective/non-selective antimicrobial supplements, and (3) selective agar plates in the assessment of sensitivity (growth) and specificity (gene detected) of the different strains (Table 2; Figure 1).

**Table 2:** Laboratory procedures testing methodologies for the detection of ESBL- and AmpC-producing *E. coli* in minced meat from cattle, pigs, and chickens.

**Method**	**Sample origin**	**Sample amount (g)**	**Pre-enrichmentbroth**	**AB supplement in borth**	**Amount of broth (ml)**	**Incubation temp (°C)**	**MacC agar with AB supplement**	**CHROM ID SMART agar**	**CHROM ID OXA agar**	**CHROM ID CARBA agar**	**Incubation temp (°C)**
MM-1	Beef	25	BPW		225	37	CRO and CTX				44
	Pork										
	Chicken meat										
MM-2	Beef	25	BPW	CRO	225	37	CRO				44
MM-3	Beef	25	BPW	CTX	225	37	CTX				44
MM-4	Beef	5	MB		45	44	CRO and CTX				44
	Pork										
MM-5	Beef	5	MB	CRO	45	44	CRO				44
	Pork										
MM-6	Beef	5	MB	CTX	45	44	CTX				44
MM-7	Beef	25	BPW		225	37		X	X	X	37
	Pork										
	Chicken meat										
MM-8	Beef	5	MB		45	44		X	X	X	37
	Pork										
MM-9	Pork	5	MB	CRO	45	44		X	X	X	37

MM, minced meat procedure; AB, antimicrobials; BPW, buffered peptone water; MB, MacConkey broth; MacC, MacConkey; CRO, ceftriaxone; CTX, cefotaxime.

The pre-enrichment assessment included the following variations: 25 g of spiked minced beef in 225 ml of Buffered Peptone Water (BPW) recommended for the food analysis of *Salmonella* spp. (Danish Standard Associaation, 2017) vs. 5 g of spiked minced beef in 45 ml of MacConkey broth (MB) [Purple, Oxoid CM0505 (CM5a)] aligned with the procedure for isolating *E. coli* in the Danish national surveillance program, DANMAP (Bager et al., 2015) (Table 2; Figure 1).

The pre-enrichment broths were with or without a supplement of third-generation cephalosporins (cefotaxime vs. ceftriaxone of 1 mg/L) and incubated for 18–24 h at either 37 or 44°C, the latter temperature was included to test the ability to inhibit the growth of background flora with reduced susceptibility to third-generation cephalosporins (Table 1; Figure 1). Plating was conducted by applying three continuous streaks onto MacConkey agar (MA) (BD Difco Ref 212123) (supplemented with 1 mg/L of either cefotaxime or ceftriaxone and incubated for 18–24 h at 44°C (Table 2; Figure 1).

The sensitivity of the six laboratory procedures was evaluated and categorized based on a semi-quantitative measure according to the load of bacterial growth on the MacConkey plates (0: No growth, 1: bacterial growth in the first streak of plating, 2: bacterial growth in the first and second streaks of plating, and 3: bacterial growth in the first, second, and third streaks of plating). For each of the six laboratory procedures, 24 bacterial colonies were selected from the selective semi-quantitative MacConkey agar plates to confirm the presence of the spiked strains by identifying the respective antimicrobial resistance genes by PCR using the primers and Polymerase chain reaction design available from the EURL-AR website (www.eurl-ar.eu). For each of the nine bacterial strains, we evaluated the performance of the laboratory methods against sensitivity by assessing the lowest detection limit (0.1, 1, 10, 100, and 1,000 CFU/g) combined with the highest bacterial growth (0–3), as well as the specificity by assessing the recovery of the spiked *E. coli* isolates harboring the following antimicrobial resistance genes: *bla*_CTX − M−1_, *bla*_SHV − 12_, *bla*_TEM − 52_, *bla*_CMY − 2_, *bla*_VIM − 1_, *bla*_NDM − 1_, *bla*_OXA − 48_, and *bla*_KPC − 2_ (Table 1).

### Minced pork

Based on the results obtained from the initial study on minced beef, the study design for minced pork was reduced to include only three laboratory procedures, MM-1, MM-4, and MM-5 (Table 2; Figure 1), the two most commonly detected third-generation cephalosporinases in food animals (*bla*_CTX − M−1_ and *bla*_CMY − 2_) and three carbapenemases (*bla*_VIM − 1_, *bla*_OXA − 48_, and *bla*_KPC − 2_).

In brief, the reduced assessment included the following variations: 25 g of spiked minced pork in 225 ml of BPW without antimicrobial supplementation and incubated for 18–24 h at 37°C (MM-1) vs. 5 g of spiked minced pork in 45 ml of MB without antimicrobial supplementation (MM-4), as well as with the supplementation of 1 mg/L of ceftriaxone (MM-5) incubated for 18–24 h at 44°C for both methods (Table 2; Figure 1). Plating was conducted using MacConkey agar supplemented with 1 mg/L of cefotaxime and/or ceftriaxone and incubated for 18–24 h at 44°C (Table 2; Figure 1). The sensitivity was evaluated and categorized based on a semi-quantitative measurement according to the load of bacterial growth. Bacterial colonies were selected from the selective semi-quantitative MacConkey agar plates to confirm the presence of the spiked strains by PCR.

The reduced procedure was tested on five *E. coli* test strains, *bla*_CTX − M−1_ (0412004714_F1), *bla*_CMY − 2_ (0412056488_F191), *bla*_VIM − 1_ (R178) (Fischer et al., 2012), *bla*_OXA − 48_ (KMU AUH), and *bla*_KPC − 2_ (17/11 RKI) (Table 1). The *E. coli* ATCC 25922 was used as the susceptible QC strain. The reason for the reduction was the highly similar results obtained previously for the tested methodologies on minced beef samples; another reason was to reduce the workload. In addition, the number of CFU concentrations tested was reduced to include the following five concentrations: 0, 1, 10, 100, and 1,000 CFU/g, since the dilution step of 1 CFU/g was sufficient to provide a precise CFU count.

All other procedures followed the procedure described above for the minced beef experiments.

### Spiking procedures of the caecal content samples

From five batches of caecal content, one batch per five *E. coli* isolates (*bla*_CTX − M−1_, *bla*_CMY − 2_, *bla*_VIM − 1_, *bla*_OXA − 48_ and one susceptible control *E. coli* ATCC 25922) (Table 1) were divided into five smaller portions of 1 g. The different portions were labeled with the isolate number and with one of the following concentrations: 0, 1, 10, 100, and 1,000 CFU/g. One of the 1 g samples labeled 0 CFU/g for each of the five portions of caecal content was selected as the negative control to ensure that the caecal content was not already contaminated with an ESBL-producing organism, an AmpC-producing organism, and CPO.

**Figure 2:**
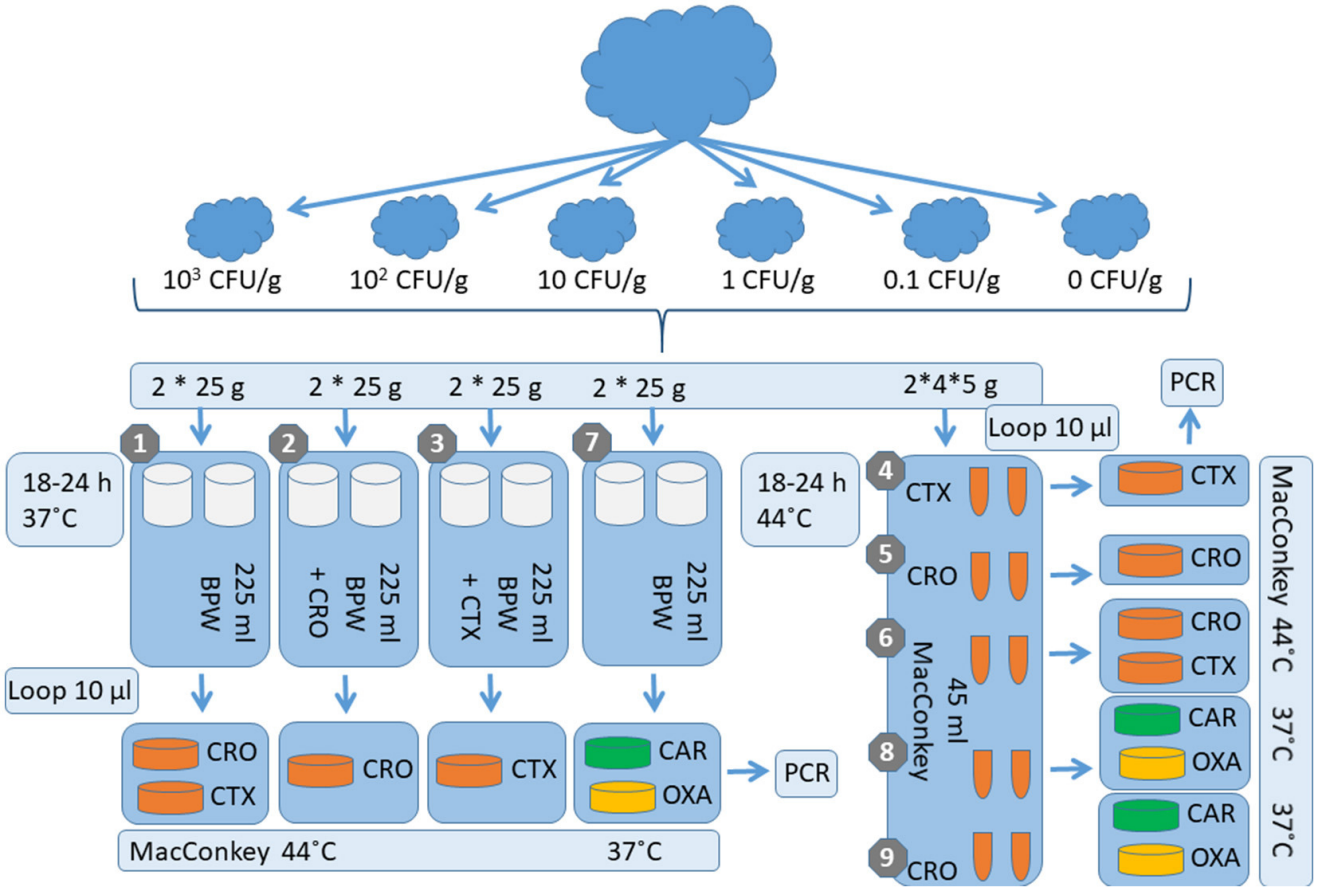
Validation scheme for the comparison of the different detection methods on caecal content. CFU/g, colony-forming unit per Gram; g, Gram; C, Celsius; h, hour; ml, milliliter; BPW, Buffered Peptone Water; CAR, ChromID CARBA medium; OXA, ChromID OXA media; MacConkey, MacConkey broth or agar; CTX, cefotaxime; CRO, ceftriaxone; PCR, Polymerase chain reaction.

The remaining four portions of 1 g each of caecal content were spiked individually with 0.010 mL of a 0.9% NaCl suspension containing each of the five individual *E. coli* test strains to contain a final concentration of 1, 10, 100, and 1,000 CFU/g per final weight of 1 g following the above procedure (Figure 2). Subsequently, the 1 g spiked caecal content samples were homogenized using a sterile spatula.

### Caecal samples from pigs and cattle

Based on the results obtained from the minced pork experiment, the study design for caecal samples from pigs and cattle focused solely on the concept of the three laboratory procedures, MM-1, MM-4, and MM-5, testing the five *E. coli* isolates (*bla*_CTX − M−1_, *bla*_CMY − 2_, *bla*_VIM − 1_, *bla*_OXA − 48_, and one susceptible control *E. coli* ATCC 25922) (Tables 1, 2; Figure 1).

In brief, the procedure included testing of 1 g of spiked caecal content from pigs and cattle in 9 ml of BPW without antimicrobial supplementation and incubating for 18–24 h at 37°C (C-1) vs. 1 g of spiked caecal content from cattle alone in 9 ml of MB without antimicrobial supplementation (C-2), as well as with the supplementation of 1 mg/L of ceftriaxone (C-3) incubated for 18–24 h at 44°C for both methods (Table 3; Figure 2). Plating was conducted using MacConkey agar supplemented with 1 mg/L of cefotaxime or ceftriaxone and incubated for 18–24 h at 44°C (Table 3; Figure 2). Sensitivity was evaluated and categorized based on a semi-quantitative measure according to the load of bacterial growth, as previously explained. Bacterial colonies were selected from the selective semi-quantitative MacConkey agar plates to confirm the presence of the spiked strains by PCR.

**Table 3:** Laboratory procedures testing methodologies for the detection of ESBL- and AmpC-producing *E. coli* from the caecal content of cattle, pigs, and chickens.

**Method**	**Sample origin**	**Sample amount (g)**	**Pre-enrichmentbroth**	**AB supplement in borth**	**Amount of broth (ml)**	**Incubation temp (°C)**	**MacC agar with AB supplement**	**CHROM ID SMART agar**	**CHROM ID OXA agar**	**CHROM ID CARBA agar**	**Incubation temp (°C)**
C-1	Cattle	1	BPW		9	37	CTX				44
	Pig										
	Chicken										
C-2	Cattle	1	MB		9	44	CTX				44
	Pig										
C-3	Cattle	1	MB	CRO	9	44	CRO				44
	Pig										
C-4	Cattle	1	BPW		9	37			X	X	37
	Pig							X	X	X	
	Chicken								X	X	
C-5	Cattle	1	MB		9	44			X	X	37
	Pig							X			
	Chicken										
C-6	Cattle	1	MB	CRO	9	44			X	X	37
	Pig							X	X	X	

C, caecal content procedure; AB, antimicrobials; BPW, buffered peptone water; MB, MacConkey broth; MacC, MacConkey; CRO, ceftriaxone; CTX, cefotaxime; MEM, meropenem.

All other procedures followed the procedure described in the minced beef experiment above.

### Chicken meat and caecal samples

Based on the results obtained from the minced beef and pork as well as caecal samples originating from cattle and pigs, one laboratory procedure was tested for minced poultry meat (MM-1) and caecal samples from chickens (C-1), respectively (Tables 2, 3; Figures 1, 2). The laboratory procedure used for both spiking and validation was identical to the point of the minced pork and caecal sample study designs testing five *E. coli* isolates (*bla*_CTX − M−1_, *bla*_CMY − 2_, *bla*_VIM − 1_, *bla*_OXA − 48_, and one susceptible control *E. coli* ATCC 25922) (Table 1) with a few exceptions.

In brief, 25 g of spiked minced chicken meat and 1 g of spiked caecal content were transferred to 225 and 9 ml of BPW without antimicrobial supplementation, respectively, and incubated for 18–24 h at 37°C (Tables 2, 3; Figures 1, 2). Plating was conducted using MacConkey agar supplemented with 1 mg/L of cefotaxime and incubated for 18–24 h at 44°C (Tables 2, 3; Figures 1, 2). The sensitivity was evaluated and categorized based on a semi-quantitative measure according to the load of bacterial growth. Bacterial colonies were selected from the selective semi-quantitative MacConkey agar plates to confirm the presence of the spiked strains by PCR.

### Validation experiments for detecting specific carbapenemase *E. coli* (part 2) — Rationale for the initial set of validation experiments

The final protocols should specifically detect bacteria with reduced susceptibility to carbapenems that are present in meat and caecal samples. Therefore, a selection of *E. coli* isolates producing various carbapenemases (including OXA-48, which is not a cephalosporinase) was included in the second part of the validation experiments.

### Spiking procedures of the minced beef, pork, and chicken meat, as well as the bovine, porcine, and chicken caecal content samples

The spiking procedures of the minced beef, pork, and chicken meat, as well as the bovine, porcine, and chicken caecal content, were performed similarly to the procedures previously described for the section on ESBL- and AmpC-producing *E. coli*.

A full study design was prepared using minced beef, pork, and chicken meat as well as bovine, porcine, and chicken caecal content as matrices, which included two laboratory procedures and an evaluation against four and five *E. coli* strains for minced meat and caecal samples, respectively.

### Minced beef and bovine caecal content samples

The design was based on the recommendation from the EFSA Scientific Opinion “on carbapenem resistance in food animal ecosystems” (EFSA BIOHAZ Panel, 2013) and on previous experience gained from a validation pilot study performed at the Federal Institute for Risk Assessment within the RESET project (http://www.reset-verbund.de/), in which different combinations of pre-enrichment (selective and non-selective), selective media (MacConkey agar supplemented with 0.125 and 0.5 mg/L of meropenem and commercial CP-selective plates), incubation conditions (37 vs. 44°C), and *E. coli* and *Salmonella* isolated from livestock used as test strains were tested as described by San José et al. (2014) and Hasman et al. (2015). From these studies, the best results (detection limit 1 CFU/g) were obtained using a non-supplemented BPW incubated at 37°C, followed by inoculation on a ChromID CARBA medium (bioMérieux), while selective pre-enrichment by MEM 0.125 mg/L was not recommended. Thus, in this present study, two laboratory procedures were conducted in duplicates and varied in terms of testing (1) different pre-enrichment broths, (2) selective/non-selective antimicrobial supplements, (3) incubation temperatures of 37 and 44°C, and (4) selective agar plates in the assessment of sensitivity (growth) and specificity (gene detected) of the different strains.

The two laboratory procedures included pre-enrichment with the following variations: 25 g of spiked minced beef in 225 ml of BPW (MM-7) vs. 5 g of spiked minced beef in 45 ml of MB without AB selection (MM-8), as well as 1 g of spiked bovine caecal content in 9 ml of BPW (C-4) vs. 1 g of spiked caecal content in 9 ml of MB without AB selection (C-5) (Tables 2, 3; Figures 1, 2). The pre-enrichment broths were incubated for 18–24 h at either 37°C (MM-7 and C-4) or 44°C [MM-8, C-5, and C-6 (C-6 was supplemented with 1 mg/L of ceftriaxone)], and the latter temperature was included to test the ability to inhibit the growth of background flora with reduced susceptibility to third-generation cephalosporins (Tables 1, 2; Figures 1, 2). Plating was conducted by applying three continuous streaks onto ChromID SMART, CARBA agar, and ChromID OXA (bioMérieux) incubated for 18–24 h at 37°C (Tables 2, 3; Figures 1, 2).

The sensitivity of the two laboratory procedures was evaluated and categorized based on the semi-quantitative measure according to the load of bacterial growth, similarly to the previous description for the section on ESBL- and AmpC-producing *E. coli* in meat and caecal samples. For both laboratory procedures, 24 bacterial colonies were selected from the selective semi-quantitative MC agar plates to confirm the presence of the spiked strains by identifying the respective antimicrobial resistance genes by PCR. For each of the bacterial strains, the performance of the laboratory methods was evaluated against sensitivity by assessing the lowest detection limit (1, 10, 100, and 1,000 CFU/g) combined with the highest bacterial growth (0–3), as well as the specificity by assessing the recovery of the spiked *E. coli* isolates harboring *bla*_VIM − 1_, *bla*_NDM − 1_, *bla*_KPC − 2_, and *bla*_OXA − 48_ (Table 1).

### Minced pork and caecal content samples

The procedure was similar to the minced beef and bovine caecal content samples, except for a few changes. In addition to the methods MM-7, MM-8, C-4, C-5, and C-6, 5 g of spiked minced pork was also mixed in 45 ml of MB supplemented with 1 mg/L of ceftriaxone (MM-9) (Table 2; Figure 1). The pre-enrichment broth (MM-9) was incubated for 18–24 h at 37°C. The plating was similar to the minced beef and bovine caecal content samples where three continuous streaks were applied solely onto the commercial selective plates ChromID CARBA agar, ChromID OXA agar, and ChromID SMART agar (bioMérieux). All plates were incubated for 18–24 h at 37°C (Table 2; Figure 1).

### Minced chicken meat and caecal content samples

Considering the results of the experiments using minced beef and pork and caecal content as matrices, the study design for minced chicken meat and caecal content samples was prepared as a continuation of these. Thus, the study design was set up to solely confirm if the selected MM-7 indicated using 25 g of spiked minced chicken meat in 225 ml of BPW had the expected sensitivity and specificity when using the commercial selective plates ChromID CARBA agar, ChromID OXA agar, and ChromID SMART agar (bioMérieux). No experiments were conducted for the caecal content (Table 2; Figure 1).

## Results

### ESBL-producing *E. coli*, AmpC-producing *E. coli*, and CP *E. coli* in minced beef

Assessing the ability of methods MM-1 to MM-3 (see Table 2; Figure 1 for details regarding all the methods) to isolate ESBL-producing *E. coli*, AmpC-producing *E. coli*, and CP *E. coli* in minced beef samples by evaluating the detection limits of the methods as well as the semi-quantitative measurements, we found no differences between using a supplemented or non-supplemented antimicrobial pre-enrichment broth; we also found no difference upon using different third-generation cephalosporins in the selective agar plates (Table 4). However, the detection limits differed slightly depending on the antimicrobial resistance gene tested with *bla*_SHV − 12_, *bla*_TEM − 52_, and _NDM − 1_ having a detection limit (1 CFU/g) that was one dilution step higher than *bla*_CTX − M−1_, *bla*_CMY − 2_, *bla*_VIM − 1_, and *bla*_KPC − 2_, all of which had a detection limit of 0.1 CFU/g (Table 4). Due to the selective principles, no growth was detected when *bla*_OXA − 48_ and the susceptible *E. coli* ATCC 25922 strain were tested (Table 4).

**Table 4:** Detection and bacterial growth of ESBL- and AmpC-producing *E. coli* in minced meat from cattle, pigs, and chickens.

**Antimicrobial classes**			**ESBLs**			**AmpC**	**CPE**				**QC**
**Method**	**Sample origin**	**Agar type**	**CTX-M-1**	**SHV-12**	**TEM-52**	**CMY-2**	**VIM-1**	**BDM-1**	**KPC-2**	**OXA-48**	**ATCC 25922**
MM-1	Beef	MacConkey+CRO	0.1 (2)	1 (2)	1 (1)	0.1 (2)	0.1 (2)	1 (1)	0.1 (3)	BD	BD
	Pork	MacConkey+CTX	1 (2)			1 (3)	1 (2)			BD	BD
	Chicken meat		0.1 (1)			0.1 (2)	0.1(2)			BD	BD
MM-2	Beef		0.1 (3)	1 (2)	1 (1)	0.1 (2)	0.1 (2)	1 (3)	0.1 (3)	BD	BD
MM-3	Beef		0.1 (3)	1 (2)	0.1 (2)	0.1 (2)	0.1 (2)	1 (2)	0.1 (2)	BD	BD
	Pork	MacConkey+CTX	0.1 (1)	1 (2)	1 (2)	1 (1)	1 (1)	1 (1)	0.1 (3)		
			1 (3)			1 (2)	1 (1)			BD	BD
MM-5	Beef		0.1 (3)	1 (2)	1 (1)	1 (1)	1 (1)	1 (1)	0.1 (2)	BD	BD
	Pork		1 (2)			1 (1)	10 (1)			BD	BD
MM-6	Beef		0.1 (3)	1 (1)	1 (1)	1 (1)	1 (1)	1 (1)	0.1 (3)	BD	BD
	Chicken meat										
MM-7	Beef	CHROM ID OXA agar	BD							1 (1)	
		CHROM ID CARBA agar	BD						0.1 (1)		
	Pork	CHROM ID OXA agar				BD	BD			1 (1)	BD
		CHROM ID CARBA agar				BD	1 (1)			BD	BD
	Chicken meat	CHROM ID OXA agar	BD			BD	BD			10 (1)	BD
		CHROM ID CARBA agar	BD			BD	BD			BD	BD
MM-8	Beef	CHROM ID OXA agar	BD							BD	
		CHROM ID CARBA agar	BD						1 (1)		
	Pork	CHROM ID OXA agar				BD	BD			1 (1)	BD
		CHROM ID CARBA agar				BD	1 (1)			BD	BD
MM-9	Pork	CHROM ID OXA agar				BD	BD			BD	BD
		CHROM ID CARBA agar				BD	1 (1)			BD	BD

The detection limit of bacteria is presented as the minimum concentration (CFU/g) in which bacteria were identified from both double-tested cultures. Bacterial growth presented in brackets is semi-quantified as growth in the first (1), second (2), or third (3) strike on the agar plate. BD, Below detection limit.

The ability of methods MM-4 to MM-6 to isolate ESBL-producing *E. coli*, AmpC-producing *E. coli*, and CP *E. coli* in minced beef samples was further assessed by applying the same evaluation criteria. This assessment also showed no differences between using a supplemented or non-supplemented antimicrobial pre-enrichment broth; it also showed no difference when using different third-generation cephalosporins in the selective agar plates. The detection limits also differed, as observed in MM-1 to MM-3, slightly depending on the antimicrobial resistance gene tested with either the same detection limit as in MM-1 to MM-3 or one level higher for *bla*_CMY − 2_ and *bla*_VIM − 1_, having an overall detection limit at 1 CFU/g for MM-4 to MM-6 (Table 4). Thus, the laboratory procedure MM-1 described by a sample size of 25 g minced beef in 225 ml of BPW without supplemented antimicrobial pre-enrichment broth was selected for the protocol.

### ESBL-producing *E. coli*, AmpC-producing *E. coli*, and CP *E. coli* in minced pork

Due to the results of the minced beef experiments, minced pork was assessed based on methods MM-1, MM-4, and MM-5 alone. The ability of all three methods to isolate ESBL- and AmpC-producing *E. coli* in minced pork samples showed similar detection limits.

Overall, the detection limit of MM-1, MM-4, and MM-5 showed a detection limit at 1 CFU/g testing *bla*_CTX − M−1_, *bla*_CMY − 2_, and *bla*_VIM − 1_, except for MM-5 testing *bla*_VIM − 1_ and having a detection limit of 10 CFU/g (Table 4). Thus, the laboratory procedure MM-1 described by a sample size of 25 g minced beef in 225 ml of BPW without supplemented antimicrobial pre-enrichment broth was selected for the protocol.

### ESBL-, AmpC-, and CP *E. coli* from caecal samples from pig and cattle

Assessing the ability of methods C-1 to C-3 to isolate ESBL- and AmpC-producing *E. coli* from caecal samples of cattle by evaluating the detection limits of the methods as well as the semi-quantitative measurements, we found no differences between using a supplemented or non-supplemented antimicrobial pre-enrichment broth; we also found no difference when using different third-generation cephalosporins in the selective agar plates (Table 5). However, the detection limits differed slightly depending on the antimicrobial resistance gene tested with *bla*_*CTX*−*M*−1_having a detection limit of 10 CFU/g (C-1 to C-3), *bla*_*CMY*−2_ having a detection limit of 10 CFU/g for C-1 to C-2 and 1 CFU/g for C-3, and *bla*_*VIM*−1_ having a detection limit of 1 CFU/g (C-1 to C-3) (Table 5). The ability of method C-1 to isolate ESBL-producing *E. coli*, AmpC-producing *E. coli*, and CP *E. coli* from caecal samples of pigs showed identical results to the C-1 method applied to the caecal samples obtained from cattle (Table 5). Due to the selective principles, no growth was detected when *bla*_OXA − 48_ and the ATCC strain were tested for both sample types (Table 5). Thus, the laboratory procedure C-1 described by a sample size of 1 g of caecal content from pigs and cattle in 9 ml of BPW without antimicrobial supplementation and incubated for 18–24 h at 37°C was selected for the protocol.

**Table 5:** Detection and bacterial growth of ESBL- and AmpC-producing *E. coli* from the caecal content of cattle, pigs, and chickens.

**Antimicrobial classes**			**ESBLs**	**AmpC**	**CPE**		**QC**
**Method**	**Sample origin**	**Agar type**	**CTX-M-1**	**CMY-2**	**VIM-1**	**OXA-48**	**ATCC 25922**
C-1	Cattle		10 (2)	10 (2)	1 (1)	BD	BD
	Pig		10 (1)	10 (1)	1 (1)	BD	BD
	Chicken		100 (1)	100 (1)	100 (1)	BD	BD
C-2	Cattle		10 (1)	10 (1)	1 (1)	BD	BD
	Pig		BD	BD	BD	BD	BD
C-3	Cattle		10 (3)	1 (2)	1 (2)	BD	BD
	Pig		BD	BD	BD	BD	BD
	Cattle	CHROM ID OXA agar	BD	BD	BD		1 (1)
		CHROM ID CARBA agar	BD	BD	1 (1)		BD
	Pig	CHROM ID OXA agar	BD	BD	BD		100 (1)
		CHROM ID CARBA agar	BD	BD	10 (1)		BD
	Chicken	CHROM ID OXA agar					
C-5		CHROM ID SMART agar					
		CHROM ID CARBA agar	BD	BD	1 (1)		BD
		CHROM ID SMART agar			BD/BD		BD/BD
		CHROM ID CARBA agar	BD	BD	BD		BD
		CHROM ID SMART agar					
		CHROM ID CARBA agar					
C-6		CHROM ID SMART agar					
		CHROM ID CARBA agar	BD	BD	1 (1)		BD
		CHROM ID SMART agar			BD/BD		BD/BDg
		CHROM ID CARBA agar	BD	BD	BD		BD
		CHROM ID SMART agar					
		CHROM ID CARBA agar					

The detection limit of bacteria is presented as the minimum concentration (CFU/g) in which bacteria were identified from both double-tested cultures. Bacterial growth presented in brackets is semi-quantified as growth in the first (1), second (2), or third (3) strike on the agar plate. BD, below detection limits. An underline values are indicate the CFU/g.

### ESBL-producing *E. coli*, AmpC- producing *E. coli*, and CP *E. coli* in minced chicken meat and caecal content

Minced chicken meat and caecal content were solely assessed based on methods MM-1 and C-1 due to the results of the previously conducted experiments. The results for the ability of method MM-1 to isolate ESBL- and AmpC-producing *E. coli* in minced chicken meat showed similar detection limits to those for the minced beef samples with 0.1 CFU/g for *bla*_CTX − M−1_, *bla*_CMY − 2_, and *bla*_VIM − 1_ (Table 4). In contrast, the ability of method C-1 to isolate ESBL- and AmpC-producing *E. coli* from caecal content showed a 1- to 2-fold difference in the detection limit (100 CFU/g for *bla*_CTX − M−1_, *bla*_CMY − 2_, and *bla*_VIM − 1_) compared to the detection limits in the caecal content originating from cattle and pigs beef samples (Table 5).

Thus, the laboratory procedures MM-1 and C-1 described by a sample size of 25 g minced chicken meat in 225 ml of BPW and 1 g of caecal content in 9 ml of BPW without antimicrobial supplementation and incubated for 18–24 h at 37°C was selected for the protocol.

Overall, despite the minute difference in the detection limit of MM-1 to MM-3 as well as C-1 to C-3, testing the minced meat and caecal content, MM-1 and C-1 were selected for the protocol. This did not include a supplemented antimicrobial pre-enrichment broth, allowing the users to further use the pre-enrichment broth, BPW, for other parts of the AMR monitoring than the specific monitoring of ESBL-producing *E. coli*, AmpC- producing *E. coli*, and CP *E. coli* from meat and caecal samples originating from cattle, pigs, and poultry, specifically, e.g., commensal *E. coli* and *Salmonella*.

### CP *E. coli* in minced beef

Assessing the ability of methods MM-7 and MM-8 to isolate CP *E. coli* in minced beef samples by evaluating the detection limits of the methods as well as the semi-quantitative measurements, we found one dilution step difference between using 25 g of minced beef in 225 ml of BPW (MM-7) and using 5 g of minced beef in 45 ml of MB (MM-8) (Table 4). Thus, the detection limit for MM-8 was one dilution step higher than that for MM-7 with a detection limit of 0.1 CFU/g for *bla*_KPC − 2_, whereas *bla*_OXA − 48_ was observed to grow (detection limit of 1 CFU/g) only when method MM-7 was applied compared to MM-8 (Table 5). Due to the selective principles, no growth was detected testing *bla*_CTX − M−1_ and the *E. coli* ATCC 25922 strain (Table 5). Thus, procedure MM-7 starting with a sample of 25 g of minced beef in 225 ml of BPW without antimicrobial supplementation and incubated for 18–24 h at 37°C was selected for the protocol.

### CP *E. coli* in minced pork

The minced pork was assessed based on methods MM-7, MM-8, and MM-9 and showed the ability of all three methods to isolate CP *E. coli* with a detection limit of 1 CFU/g for *bla*_VIM − 1_ and *bla*_OXA − 48_ (no growth in MM-9, expected considering its phenotype) (Table 5). Thus, the detection limit for MM-8 was one dilution step higher than that for MM-7. Due to the selective principles, no growth was detected when *bla*_CTX − M−1_ and the susceptible *E. coli* ATCC 25922 strain were tested (Table 5). Thus, similar to beef, the laboratory procedure MM-7, starting with a sample of 25 g minced pork in 225 ml of BPW without supplemented antimicrobial pre-enrichment broth, was selected to be included in the protocol.

### CP *E. coli* from the caecal samples of pig and cattle

When assessing the ability of methods C-4 to C-6 to isolate CP *E. coli* from the caecal samples of pig and cattle by evaluating the detection limits of the methods as well as the semi-quantitative measurements, no growth was obtained with either method C5 (unexpected) or C-6 (expected considering the resistance phenotype of the isolate). Similarly, no growth was observed when assessing the ability of methods C-5 and C-6 by testing *bla*_VIM − 1_ for the caecal samples of pigs; the detection limit for caecal samples obtained from cattle was 1 CFU/g (Table 5). C-4 was assessed to have the ability to isolate CP *E. coli* from caecal samples with a detection limit of 1 CFU/g for both *bla*_VIM − 1_ and *bla*_OXA − 48_ obtained from cattle, as opposed to a detection limit of 10 CFU/g (*bla*_VIM − 1_) and 100 CFU/g (*bla*_OXA − 48_) from pigs (Table 5). Due to the selective principles, no growth was detected when *bla*_CTX − M−1_, *bla*_CMY − 2_, and the susceptible *E. coli* ATCC 25922 strain were tested (Table 4). Thus, laboratory procedure C-4 described by a sample size of 1 g caecal content in 9 ml of BPW without supplemented antimicrobial pre-enrichment broth was selected for the protocol.

### CP *E. coli* in minced chicken meat

The minced chicken meat was assessed for the ability to detect CP *E. coli* using method MM-7 due to the results of the previously conducted experiments. The ability of method MM-7 to isolate CP *E. coli* in minced chicken meat showed no growth for *bla*_VIM − 1_ (See Discussion section), whereas it showed a detection limit one dilution step higher than that for minced beef and pork with 10 CFU/g for *bla*_OXA − 48_ (Table 4).

Thus, the laboratory procedure MM-7, which started with a sample of 25 g minced chicken meat in 225 ml of BPW without antimicrobial supplementation that was incubated for 18–24 h at 37°C was selected for the protocol.

Overall, after testing the minced meat and caecal content, MM-7 and C-4 were selected for the protocol. This did not include a supplemented antimicrobial pre-enrichment broth, allowing the users to further use the pre-enrichment broth and BPW for other parts of the monitoring than the specific monitoring of ESBL-producing *E. coli*, AmpC-producing *E. coli*, and CP *E. coli* from meat and caecal samples originating from cattle, pigs, and poultry, specifically, e.g., commensal *E. coli* and *Salmonella*.

## Discussion

To ensure a harmonized approach to monitoring, standardized laboratory protocols are essential. When considering which laboratory protocol is to be used for monitoring a specific pathogen, the advantages and disadvantages of the protocol must be accounted for, such as test sensitivity and specificity. Additionally, laboratory and infrastructure practicalities as well as any economic aspects must also be considered. For example, whether or not a pre-enrichment broth for a given laboratory procedure targeting a specific phenotype and species will also be useful for other species must be considered. In general, a selective pre-enrichment step increases sensitivity whenever an antimicrobial-resistant bacteria of concern is present in extremely low numbers. The inclusion of a selective pre-enrichment step, however, may facilitate horizontal gene transfer and may limit the use of the broth for detecting other bacteria under surveillance, even though this principle has been challenged recently (Lopatkin et al., 2016).

The preparatory work when developing the two EURL-AR protocols for meat (protocol 1) and caecal (protocol 2) samples, respectively, of the specific monitoring of ESBL- and AmpC-producing *E. coli* (part 1) and CP *E. coli* (part 2) originating from cattle, pigs, and poultry in the EU used in the Implementing Decision on the monitoring and reporting of antimicrobial resistance in zoonotic and commensal bacteria (2013/652/EU) (European Union, 2013), which was repealed in 2021 by the Implementing Decision (EU) 2020/1729 of 17 November 2020 (European Union 2020), showed that method MM-1 (protocol 1) and C-1 (protocol 2) were the most optimal protocols among the tested approaches for minced meat and caecal content sample, with a detection limit ranging from 0.1 to 100 CFU/g, depending on the matrix and test strains. Of note, the detection limits are presented in the most conservative way to include the minimum value for both double-tested samples. Hence, if the detection limit for one sample is 10 CFU/g and the other is 1 CFU/g, only the highest concentration (10 CFU/g) is presented.

Both MM-1 and C-1 included a non-selective pre-enrichment broth based on BPW and subsequent plating on selective agar plates, MacConkey agar supplemented with 1 mg/L cefotaxime for the detection of ESBL- and AmpC-producing *E. coli* and Chrom ID SMART (Chrom ID CARBA and OXA) for CP *E. coli*. ChromID CARBA (bioMérieux) is known to have the highest specificity (76%) and sensitivity (96%), and in our experience, this medium performs better as single plates rather than in combined half-plates with Chrom ID OXA in the Chrom ID SMART plates. An in-house media (e.g., MacConkey agar) supplemented with a carbapenem or a chromogenic medium could also be applied as a selective agar for isolating CP *E. coli*. These chromogenic media, however, generally show difficulties when detecting *bla*_OXA − 48_ producers due to the low carbapenem MICs. Therefore, ChromID OXA (bioMérieux) was developed for the specific detection of *bla*_OXA − 48_ producers as it prohibits the growth of class A and B carbapenemases. However, it should be used in combination with another selective medium (EFSA BIOHAZ Panel, 2013), e.g., ChromID SMART (bioMérieux).

Recently, a similar protocol, the Tricycle Protocol (Jacob et al., 2020; World Health Organization, 2021), was published as a screening tool in the global surveillance of ESBL-producing *E. coli*; it is based on the principle of MacConkey agar with higher selection pressure (cefotaxime 4 mg/L). In our experience, a cefotaxime concentration at 4 mg/L is too high to detect some enzymes, such as *bla*_TEM − 20_, *bla*_TEM − 52_, and *bla*_CMY − 2_; thus, as of this study, we would propose the use of a concentration of 1 mg/L cefotaxime as screening cut-off, as also recommended by EUCAST to detect ESBL- and AmpC-producing *E. coli* (https://www.eucast.org/fileadmin/src/media/PDFs/EUCAST_files/Resistance_mechanisms/EUCAST_detection_of_resistance_mechanisms_170711.pdf). The Tricycle Protocol further addressed the need for a standardized MacConkey recipe, as MacConkey agar varies substantially between manufacturers, which may influence the growth of *E. coli* (Jacob et al., 2020).

It would be of utmost importance to perform follow-up research based on the results of the current study, with further investigation on the test sensitivity and specificity of the two suggested laboratory protocols concerning media and selection pressure.

In developing this protocol, the laboratory testing of the different methodologies was designed as a “cascade assessment,” where the testing and variables were reduced for the next experiment based on the results of the preceding experiment, e.g., the minced pork experiment was reduced based on the outcome of the minced beef experiment. This was implemented due to the lack of time, enabling the publication of the protocols before the legislation came into force. Nonetheless, we observed during the experiment that most methods detected the spiked test strain, with the exception of where growth should not be expected, e.g., genes conferring resistance to third-generation cephalosporins should not grow on media containing carbapenems Chrom ID agar, and strains containing *bla*_OXA − 48_ should not grow on Chrom ID CARBA as opposed to Chrom ID OXA and vice versa for strains harboring *bla*_VIM − 1_, for example. In a single case in the method for detecting CP *E. coli*, one of the test strains (*bla*_VIM − 1_ producing *E. coli* strain R178) did not grow. This might be related to the specific strain harboring the *bla*_VIM − 1_, which had an extremely low MIC for carbapenems, e.g., in this study, ertapenem MIC = 0.06, imipenem MIC = 0.5, and meropenem MIC = 0.12 or the strain harboring the *bla*_VIM − 1_, which originated from a different animal species than the matrix to which the spiked strain belonged. Multiple times, when preparing reference material for the EURL-AR iterations of the External Quality Assessment schemes on the selective isolation of presumptive ESBL-producing *E. coli*, AmpC- producing *E. coli*, and CP *E. coli* from meat and caecal samples (Matrix EQAS) (https://www.eurl-ar.eu/reports.aspx), we made similar observations. In this study, we observed that*bla*_VIM − 1_, e.g., present in a strain originating from a chicken sample did not survive well the spiking into a matrix of pig caeca. Based on this, we speculated whether the caeca in these examples contained some sort of inhibitor responsible for killing the strain isolated from the caecum of a different animal species (no data available). Thus, we decided to still recommend the C-1 procedure despite the lack of growth by the *bla*_VIM − 1_ strain based on the results obtained by a previous study conducted within the RESET Project (San José et al., 2014).

During the experiments, we also observed that, for the meat samples, the BPW methods tended to generate more background growth of accompanying flora than the MacConkey methods. This was opposed to the MacConkey methods showing no growth in all of the pig caecal samples when pre-incubating in MB. This interesting and limiting observation was perhaps due to the presence of bile salts in the caecal samples from pigs combined with the bile salts in the MB, which could have killed the bacteria. Nonetheless, this observation was considered and judged to be a greater limitation than background growth with the application of the BPW. It is noteworthy that another issue that could contribute to the increased psychotropic background flora could be related to the time in which the samples were maintained in the refrigerator before analysis. In our experience (data not shown), those samples spiked after a couple of days and presented higher background flora mainly the previous study conducted within the RESET Project (San José et al., 2014), which must be considered when investigating the samples during the monitoring program.

An advantage of selecting a method based on BPW (MM-1 and C-1) was the opportunity taken by many NRLs to use the BPW for other parts of the Implementing Decision (EU) 2020/1729 of 17 November 2020 (European Union, 2020). A recent questionnaire survey with participation from 34 European National Reference Laboratories and affiliated laboratories, representing 32 countries, investigated the extent to which the pre-enrichment broth for ESBL was re-used in the surveillance of other pathogens. The survey results showed that the pre-enrichment broth for caecal content was used to identify *Salmonella*, commensal *E. coli*, and enterococci by 66, 32, and 50%, respectively, of the laboratories. Similarly, the pre-enrichment broth from meat samples was used to identify *Salmonella*, commensal *E. coli*, and enterococci by 71, 58%, and 50%, respectively, of the laboratories (https://www.eurl-ar.eu/CustomerData/Files/Folders/25-resourcer/593_survey-eurl-ar-esblprotocol.pdf). The numbers for enterococci, however, represent relatively few laboratories testing for enterococci regularly for both caecal contents (10 laboratories) and meat samples (six laboratories).

Several considerations and limitations were introduced while developing the protocols, which may have affected the outcome. A cephalosporin, such as cefotaxime or ceftriaxone, could be added to the pre-enrichment buffer to potentially enrich ESBL/AmpC producers before plating. This selective pre-enrichment could, however, exclude some *bla*_OXA − 48_-group producers, if a cephalosporinase-encoding gene is not harbored simultaneously. Furthermore, the inclusion of a cephalosporin or carbapenem in the media could trigger horizontal gene transfer during pre-enrichment (Liu et al., 2019). Similarly, a low concentration of a carbapenem could have been used as a supplement to the pre-enrichment broth; however, this might have resulted in the growth of a substantial part of background flora, such as *Pseudomonas* spp., with intrinsic resistance to certain carbapenems, thereby making the isolation and detection of CPEs challenging. For the detection of CPE in extremely low numbers for control efforts, a selective pre-enrichment including a carbapenem in low concentration (e.g., meropenem 0.125 mg/L) may be required. This approach could potentially increase sensitivity while excluding any presence of OXA-48 and similar producers, as well as other isolates expressing low resistance to carbapenems close to the screening cut-off/ECOFFs, as the *bla*_VIM − 1_ isolate used in this study (Fischer et al., 2012).

Considering that the presence of CP *E. coli* is still rare in food-producing animals and the meat thereof and that the methodology proposed could have a low detection limit for some types of carbapenemases, for almost a decade, after the implementation in 2014, the isolation protocol has facilitated the detection of CP *E. coli* from meat and caecal samples in a few EU MSs (Borowiak et al., 2017; Irrgang et al., 2017, 2020a,b; Madec et al., 2017; Diaconu et al., 2020; Garcia-Graells et al., 2020; Bortolaia et al., 2021; Carfora et al., 2022; European Food Safety Authority European Centre for Disease Prevention Control, 2023). Thus, the EURL-AR protocols have been proven to fulfill the purpose of facilitating the monitoring and reporting of antimicrobial resistance in zoonotic and commensal bacteria according to the Implementing Decisions 2013/652/EU and 2020/1729, for the specific isolation of ESBL-producing *E. coli*, AmpC-producing *E. coli*, and CP *E. coli* from meat and caecal samples. The protocols are contributing to the detection of this slow but emerging threat in the food chain in a harmonized way.

## Conclusion

We evaluated and validated several laboratory procedures based on EFSA recommendations to provide two isolation protocols for fresh meat and caecal samples, respectively, used for the EU-specific monitoring of ESBL-producing *E. coli*, AmpC-producing *E. coli*, and CP *E. coli*.

The laboratory procedures tested revealed that the most sensitive and specific methodology was a procedure based on a BPW pre-enrichment step, followed by inoculation onto MacConkey agar supplemented with cefotaxime for detecting ESBL- and AmpC-producing *E. coli* and Chrom ID for detecting CP *E. coli*. The protocol also allows for the BPW pre-enrichment to be used for other parts of the EU monitoring due to non-supplementation with antimicrobials. For specific field and control studies for the detection of CP *E. coli*, the sensitivity and specificity might be enhanced by supplementing the pre-enrichment with carbapenem, although this will not be proficient for detecting *bla*_OXA − 48_-like enzymes. The protocol continues to be used by all EU MSs for the present monitoring period (2021–2027).

## Data availability statement

The original contributions presented in the study are included in the article/supplementary material, further inquiries can be directed to the corresponding author.

## Author contributions

HH, LC, BG, VB, and YA designed the study and discussed the results gained. CS and HN conducted the lab experiments and generated the data. JK and SP revised the protocol. RH and MF drafted the first version of the manuscript whereas HH and BG assisted with the last version the manuscript as well as revised the manuscript. All authors have read and approved the final manuscript.

## Author disclaimer

BG is currently employed by the European Food Safety Authority (EFSA) in its BIOHAW Unit, which provides scientific and administrative support to EFSA's scientific activities in the area of Microbial Risk Assessment. The positions and opinions presented in this manuscript are those of the authors alone and are not intended to represent the views or scientific works of EFSA. Some of the studies performed at DTU Food run in parallel with those from the RESET project at the Federal Institute for Risk Assessment, where BG was working at that time.

## References

[B1] Aworh M. K. Ekeng E. Nilsson P. Egyir B. Owusu-Nyantakyi C. Hendriksen R. S. (2022). Extended-spectrum ÃÝ-lactamase-producing escherichia coli among humans, beef cattle, and abattoir environments in Nigeria. Front. Cell Infect. Microbiol. 12, 869314. 10.3389/fcimb.2022.869314 35463650 PMC9021871

[B2] Bager F. Birk T. Borck Høg B. Jensen L. B. Jensen A. N. de Knegt L. (2015). DANMAP 2014 - Use of Antimicrobial Agents and Occurrence of antImicrobial Resistance in Bacteria From Food aniMals, Food and Humans in Denmark. DANMAP. Available online at: http://www.danmap.org/~/media/Projekt%20sites/Danmap/DANMAP%20reports/DANMAP%202014/Danmap_2014.ashx (accessed July 31, 2023).

[B3] Borowiak M. Szabo I. Baumann B. Junker E. Hammerl J. A. Kaesbohrer A. . (2017). VIM-1-producing Salmonella Infantis isolated from swine and minced pork meat in Germany. J. Antimicrob. Chemother. 72, 2131–2133. 10.1093/jac/dkx101 28369508

[B4] Bortolaia V. Ronco T. Romascu L. Nicorescu I. Milita N. M. Vaduva A. M. . (2021). Co-localization of carbapenem (blaOXA-162) and colistin (mcr-1) resistance genes on a transferable IncHI2 plasmid in *Escherichia coli* of chicken origin. J. Antimicrob. Chemother. 76, 3063–3065. 10.1093/jac/dkab285 34392339 PMC8521400

[B5] Carattoli A. (2008). Animal reservoirs for extended spectrum beta-lactamase producers. Clin. Microbiol. Infect. 14 (Suppl. 1), 117–123. 10.1111/j.1469-0691.2007.01851.x 18154535

[B6] Carfora V. Diaconu E. L. Ianzano A. Di M. P. Amoruso R. Dell'Aira E. . (2022). The hazard of carbapenemase (OXA-181)-producing *Escherichia coli* spreading in pig and veal calf holdings in Italy in the genomics era: risk of spill over and spill back between humans and animals. Front. Microbiol. 13, 1016895. 10.3389/fmicb.2022.1016895 36466661 PMC9712188

[B7] Danish Standard Associaation. (2017). ISO 6579-1:2017 Microbiology of the food chain — Horizontal method for the detection, enumeration and serotyping of Salmonella — Part 1: Detection of Salmonella sp. Available online at: https://www.iso.org/standard/56712.html (accessed July 31, 2023).

[B8] DANMAP (2012). Use of Antimicrobial Agents and Occurrence of Antimicrobial Resistance in Bacteria From Food Animals, Food and Humans in Denmark. Avaialble online at: https://www.danmap.org/reports/2012 (accessed July 31, 2023).

[B9] Dantas P. J. Ferreira H. M. N. (2020). Extended-spectrum beta-lactamase (ESBL)-producing Enterobacteriaceae in cattle production - a threat around the world. Heliyon 6, e03206. 10.1016/j.heliyon.2020.e03206 32042963 PMC7002838

[B10] Diaconu E. L. Carfora V. Alba P. Di M. P. Stravino F. Buccella C. . (2020). Novel IncFII plasmid harbouring blaNDM-4 in a carbapenem-resistant Escherichia coli of pig origin, Italy. J. Antimicrob. Chemother. 75, 3475–3479. 10.1093/jac/dkaa,374 32835381 PMC7662189

[B11] EFSA BIOHAZ Panel (2013). Scientific Opinion on Carbapenem resistance in food animal ecosystems. EFSA J. 11, 3501. 10.2903/j.efsa.2013.3501

[B12] EFSA Panel on Biological Hazards (2011). Scientific Opinion on the public health risks of bacterial strains producing extended-spectrum β-lactamases and/or AmpC β-lactamases in food and food-producing animals. EFSA J. 9, 2322. 10.2903/j.efsa.2011.2322

[B13] European Food Safety Authority and European Centre for Disease Prevention and Control (2023). The European Union Summary Report on Antimicrobial Resistance inzoonotic and indicator bacteria from humans, animals and food in 2020/2021. EFSA J. 21, 7867. 10.2903/j.efsa.2023.7867 36891283 PMC9987209

[B14] European Union (2013). Commission Implementing Decision of 12 November 2013 on the monitoring and reporting of antimicrobial resistance in zoonotic and commensal bacteria (2013/652/EU). Off J. Eur Union. 26–39.

[B15] European Union (2020). Commission Implementing Decision (EU) 2020/1729 of 17 November 2020 on the monitoring and reporting of antimicrobial resistance in zoonotic and commensal bacteria and repealing Implementaing Decision 2013/652/EU. (2020/1729/EU). Off J Eur Union 8–21.

[B16] Fischer J. Rodriguez I. Schmoger S. Friese A. Roesler U. Helmuth R. . (2012). *Escherichia coli* producing VIM-1 carbapenemase isolated on a pig farm. J. Antimicrob. Chemother. 67, 1793–1795. 10.1093/jac/dks108 22454489

[B17] Fischer J. Rodriguez I. Schmoger S. Friese A. Roesler U. Helmuth R. . (2013). Salmonella enterica subsp. enterica producing VIM-1 carbapenemase isolated from livestock farms. J. Antimicrob. Chemother. 68, 478–480. 10.1093/jac/dks393 23034713

[B18] Fischer J. San J. M. Roschanski N. Schmoger S. Baumann B. Irrgang A. . (2017). Spread and persistence of VIM-1 Carbapenemase-producing Enterobacteriaceae in three German swine farms in 2011 and 2012. Vet. Microbiol. 200, 118–123. 10.1016/j.vetmic.2016.04.026 27234907

[B19] Garcia-Graells C. Berbers B. Verhaegen B. Vanneste K. Marchal K. Roosens N. H. C. . (2020). First detection of a plasmid located carbapenem resistant bla(VIM-1) gene in *E. coli* isolated from meat products at retail in Belgium in 2015. Int. J. Food Microbiol. 324, 108624. 10.1016/j.ijfoodmicro.2020.108624 32302878

[B20] Guerra B. Fischer J. Helmuth R. (2014). An emerging public health problem: acquired carbapenemase-producing microorganisms are present in food-producing animals, their environment, companion animals and wild birds. Vet. Microbiol. 171, 290–297. 10.1016/j.vetmic.2014.02.001 24629777

[B21] Hamza E. Dorgham S. M. Hamza D.A. (2016). Carbapenemase-producing Klebsiella pneumoniae in broiler poultry farming in Egypt. J. Glob. Antimicrob. Resist. 7, 8–10. 10.1016/j.jgar.2016.06.004 27530998

[B22] Hasman H. Agers ø Y. Cavaco L. Svendsen C. A. Nielsen H. San Jose M. . (2015). Evaluation of Methods for Enrichment of Carbapenemase-Producing E. coli in Pork Meat and Cecal Samples of Porcine and Bovine Origin: EV0266. Abstract From 25th European Congress of Clinical Microbiology and Infectious Diseases. Copenhagen: ECCMID.

[B23] Irrgang A. Fischer J. Grobbel M. Schmoger S. Skladnikiewicz-Ziemer T. Thomas K. . (2017). Recurrent detection of VIM-1-producing *Escherichia coli* clone in German pig production. J. Antimicrob. Chemother. 72, 944–946. 10.1093/jac/dkw479 28007897 PMC5400094

[B24] Irrgang A. Pauly N. Tenhagen B. A. Grobbel M. Kaesbohrer A. Hammerl A.J.A. (2020a). Spill-over from public health? First detection of an OXA-48-producing *Escherichia coli* in a German Pig Farm. Microorganisms 8, 855. 10.3390/microorganisms8060855 32517147 PMC7356166

[B25] Irrgang A. Tausch S. H. Pauly N. Grobbel M. Kaesbohrer A. Hammerl J.A. (2020b). First detection of GES-5-producing *Escherichia coli* from livestock-an increasing diversity of carbapenemases recognized from german pig production. Microorganisms 8, 1593. 10.3390/microorganisms8101593 33081194 PMC7602714

[B26] Irrgang A. Tenhagen B. A. Pauly N. Schmoger S. Kaesbohrer A. Hammerl J.A. (2019). Characterization of VIM-1-producing *E. coli* isolated from a German fattening pig farm by an improved isolation procedure. Front. Microbiol. 10, 2256. 10.3389/fmicb.2019.02256 31632372 PMC6779854

[B27] Jacob M. E. Keelara S. Idara-Kane A. Matheu A. Jr. Fedorka-Cray P.J. (2020). Optimizing a screening protocol for potential extended-spectrum Î^2^-lactamase *Escherichia* coli on macconkey agar for use in a global surveillance program. J. Clin. Microbiol. 58, e01039–e01019. 10.1128/JCM.01039-19 32434784 PMC7448649

[B28] Kock R. Niels-Haardt I. Becker K. Mellmann A. Friedrich A. W. Mevius D. . (2018). Carbapenem-resistant Enterobacteriaceae in wildlife, food-producing, and companion animals: a systematic review. Clin, Microbiol. Infect. 24, 1241–1250. 10.1016/j.cmi.2018.04.004 29654871

[B29] Li J. Bi Z. Ma S. Chen B. Cai C. He J. . (2019). Inter-host transmission of carbapenemase-producing *Escherichia coli* among humans and backyard animals. Environ. Health Perspect. 127, 107009. 10.1289/EHP5251 31642700 PMC6910777

[B30] Liu G. Bogaj K. Bortolaia V. Olsen J. E. Thomsen L.E. (2019). Antibiotic-induced, increased conjugative transfer is common to diverse naturally occurring ESBL plasmids in *Escherichia coli*. Front. Microbiol. 10, 2119. 10.3389/fmicb.2019.02119 31552012 PMC6747055

[B31] Lopatkin A. J. Huang S. Smith R. P. Srimani J. K. Sysoeva T. A. Bewick S. . (2016). Antibiotics as a selective driver for conjugation dynamics. Nat. Microbiol. 1, 16044. 10.1038/nmicrobiol.2016.44 27572835 PMC5010019

[B32] Madec J. Y. Haenni M. Nordmann P. Poirel L. (2017). Extended-spectrum Î^2^-lactamase/AmpC- and carbapenemase-producing Enterobacteriaceae in animals: a threat for humans? Clin. Microbiol. Infect. 23, 826–833. 10.1016/j.cmi.2017.01.013 28143782

[B33] Mughini-Gras L. Dorado-Garcia A. van D. E. van den B. G. Dierikx C. M. Bonten M. J. M. . (2019). Attributable sources of community-acquired carriage of Escherichia coli containing Î^2^-lactam antibiotic resistance genes: a population-based modelling study. Lancet Planet Health 3, e357–e369. 10.1016/S2542-5196(19)30130-5 31439317

[B34] Poirel L. Walsh T. R. Cuvillier V. Nordmann P. (2011). Multiplex PCR for detection of acquired carbapenemase genes. Diagn. Microbiol. Infect. Dis. 70, 119–123. 10.1016/j.diagmicrobio.2010.12.002 21398074

[B35] San José M. Hasman H. Fischer J. Agerso Y. Schmoger S. Jahn S. . (2014). Evaluation of Methods for Detection of VIM-1-Carbapenemase-Producing Enterobacteriaceae in Bovine Minced Meat. (ECCMID 2014 - eP334). Barcelona: ECCMID.

[B36] Shi X. Li Y. Yang Y. Shen Z. Cai C. Wang Y. . (2021). High prevalence and persistence of carbapenem and colistin resistance in livestock farm environments in China. J. Hazard Mater. 406, 124298. 10.1016/j.jhazmat.2020.124298 33168321

[B37] World Health Organization (2021). WHO Integrated Global Surveillance on ESBL-Producing E. coli Using a “One Health” Approach: Implementation and Opportunities. World Health Organization. Available online at: https://apps.who.int/iris/handle/10665/340079 (accessed July 31, 2023).

